# Antimicrobial susceptibility patterns in Jalisco, Mexico: a snapshot of regional resistance (2021-2023)

**DOI:** 10.3389/fcimb.2026.1866897

**Published:** 2026-09-22

**Authors:** Braulio Josué Méndez-Sotelo, Marco Antonio Delaye-Martínez, Adolfo Gómez-Quiroz, Sara Alejandra Aguirre-Díaz, Rodrigo Escobedo-Sánchez, José Manuel Ramírez-Rodríguez, Alba Guadalupe Juárez-Lomelí, Claudia Lara-Rangel, Constanza Otero, Nils Ramírez, Luisina Carozza-Renaud, Ixchel Magaña-Matienzo, Elvira Garza-González, Rafael Franco-Cendejas, Luis Esaú López-Jácome

**Affiliations:** 1 Infectología, Hospital General León, León, Guanajuato, Mexico; 2 Plan de Estudios Combinados en Medicina (PECEM), Facultad de Medicina, Universidad Nacional Autónoma de México, Mexico City, Mexico; 3 Departamento de Infectología, Instituto Nacional de Cancerología, Mexico City, Mexico; 4 Laboratorio de Microbiología Clínica. Hospital Civil de Guadalajara Fray Antonio Alcalde, Guadalajara, Jalisco, Mexico; 5 Infectología, Hospital Civil de Guadalajara Fray Antonio Alcalde, Guadalajara, Jalisco, Mexico; 6 Unidad de Vigilancia Epidemiológica, Hospital San Javier, Guadalajara, Jalisco, Mexico; 7 Laboratorio, Hospital México Americano, Guadalajara, Jalisco, Mexico; 8 Real-World Evidence – Epidemiology & Clinical Data Management Latin America, Central America and The Caribbean; 9 Urgencias, Hospital General Dr. Manuel Gea González, Mexico City, Mexico; 10 Departamento de Bioquímica y Medicina Molecular, Facultad de Medicina., Universidad Autónoma de Nuevo León, Monterrey, Nuevo León, Mexico; 11 Subdirección de Investigación Biomédica, Instituto Nacional de Rehabilitación Luis Guillermo Ibarra Ibarra, Mexico City, Mexico; 12 Laboratorio de Microbiología Clínica, Instituto Nacional de Rehabilitación Luis Guillermo Ibarra Ibarra, Mexico City, Mexico; 13 Departamento de Biología, Facultad de Química, Universidad Nacional Autónoma de México, Mexico City, Mexico

**Keywords:** antimicrobial resistance (AMR), ESKAPE pathogens, hospitals, Mexico, microbial

## Abstract

**Background:**

Antimicrobial resistance (AMR) is a major global public health threat, responsible for an estimated 4.9 million deaths worldwide and associated with increased morbi-mortality, and healthcare costs. The burden is particularly high in hospital settings, where multidrug-resistant pathogens, including ESKAPE group, are prevalent. However, in Mexico, epidemiological data remain limited. This study aimed to determine the prevalence and distribution of AMR among clinically relevant bacterial pathogens isolated in three hospitals in Guadalajara, Jalisco, Mexico, and to compare resistance patterns across hospital wards and clinical specimen types.

**Methods:**

A retrospective multicenter study was conducted from January 2021 to December 2023. Bacterial identification and susceptibility tests were performed using VITEK 2 and MicroScan WalkAway systems. Results were analyzed using WHONET and interpreted according to CLSI criteria. Clinical specimens included urine, blood, biopsies, abscesses, lower respiratory tract (LRT) specimens, and sterile fluids. Species analyzed included, *Enterococcus* spp., *Staphylococcus aureus, Klebsiella pneumoniae, Acinetobacter baumannii, Pseudomonas aeruginosa, Enterobacter baumannii and Escherichia coli.* Statistical comparisons were performed using R Statistical Software Studio.

**Results:**

A total of 13,336 clinical isolates were analyzed, with *E. coli* (n= 5,737) and *K. pneumoniae* (n= 1,738) as the most predominant pathogens. Urine samples were the most frequently collected (n= 4,247). Bloodstream isolates exhibited the highest resistance levels for *E. coli* and *K. pneumoniae*. Non-fermenting pathogens showed high resistance in LRT isolates, especially for *P. aeruginosa* and *A. baumannii*, with elevated carbapenem resistance. Resistance levels were higher the ICU, particularly for *K. pneumoniae*, *E. cloacae*, *P. aeruginosa*, and A. *baumannii*. In contrast, the non-ICU settings showed a greater prevalence of ESBL-producing *E. coli*.

**Conclusions:**

This study reveals substantial AMR burden in Guadalajara, with particularly high levels among bloodstream pathogens, non-fermenters in LRT specimens, and carbapenem-resistant *K. pneumoniae* and *A. baumannii*. Resistance patterns varied across hospital wards and specimen types, with higher resistance in ICU. These findings contribute valuable regional data to national AMR monitoring efforts.

## 1 Introduction

Antimicrobial resistance (AMR) is a natural process that has been dramatically accelerated by the extensive use of antimicrobial agents in human and animal medicine, as well as in agriculture (McEwen and Collignon, 2018; Murray et al., 2022; Center for Disease C and Prevention, 2024). AMR represents a significant threat to the effective prevention and treatment of infectious diseases (World Health O; Chinemerem Nwobodo et al., 2022).

In 2017, the World Health Organization (WHO) published a global list of priority pathogens categorized as critical, high, and medium priority, for which new antimicrobial strategies and drug development are urgently needed (World Health Organization, 2017). Among these, the ESKAPE group, comprising vancomycin-resistant *Enterococcus* spp., methicillin-resistant *Staphylococcus aureus* (MRSA), third- and fourth-generation cephalosporin-resistant, carbapenem-resistant *Klebsiella* spp., as well as carbapenem-resistant *Acinetobacter baumannii* and *Pseudomonas aeruginosa*, represents a major concern due to its ability to “escape” the effects of antimicrobial agents (World Health Organization, 2017; Kelly et al., 2024). In addition, *Escherichia coli*, one of the most common causes of community- and healthcare-associated infections, and *Stenotrophomonas maltophilia*, characterized by intrinsic multidrug resistance, have been included in an expanded group referred to as E2SKAPES (World Health O; Xuemei et al., 2019; De Oliveira et al., 2020).

Globally, AMR has been associated with approximately 4.9 million deaths. *E. coli, S. aureus, K. pneumoniae, A. baumannii*, and *Streptococcus pneumoniae* accounted for an estimated 250,000 deaths in 2019 (Murray et al., 2022). Notably, *E. coli* is consistently reported as the most frequently isolated pathogen in both community and hospital settings, frequently exhibiting extended-spectrum β-lactamase (ESBL) production (Bezabih et al., 2022; Fernandez et al., 2023; Aiesh et al., 2024). At the same time, non-fermenting Gram-negative bacteria, particularly *A. baumannii*, have emerged as critical threats due to their high levels of resistance to carbapenems and other last-line antibiotics (Najafabadi and Soltani, 2024; Thacharodi et al., 2024; De Souza et al., 2025).

Prior to COVID-19 pandemic, carbapenem resistance in *K. pneumoniae* showed significant geographic variability, remaining around 2% in the United States, but exceeded 50% in countries such as Belarus, Georgia, Greece, Moldova, Russia and Ukraine. In Latin America reported rates ranged from 0.8 to 12.7% (William and Cesar, 2024). While for *A. baumannii*, after COVID-19 resistance to carbapenems, specifically meropenem, was higher as 89.2% in Egypt (Abdelaziz Abdelmoneim et al., 2024) and 97.8% for Türkiye (Çapar et al., 2025), on the other hand, for *P. aeruginosa* resistance to imipenem rose 51.5% (18.8% Pre-pandemic) (Çapar et al., 2025). However, increasing resistance among both Enterobacterales and non-fermenting bacteria highlights the need for robust local surveillance data to better understand evolving resistance patterns (Lee et al., 2024; Ge et al., 2026).

In response to the growing threat of AMR, the WHO published the Global Action Plan in 2015, emphasizing the importance of strengthening epidemiological surveillance (World Health Organization, 2015). In line with these efforts, Mexico launched the National Strategy against AMR in 2018 to promote research in this field and guide national actions. Within this context, the present study aimed to determine the frequency and distribution of AMR among clinically relevant pathogens, including *E. coli, K. pneumoniae, P. aeruginosa, A. baumannii* and Gram-positive organisms, in healthcare institutions in central Mexico.

## 2 Material and methods

### 2.1 Data collection and study design

A retrospective, observational, multicenter study was conducted in three hospitals located in Jalisco, Mexico, from January 2021 to December 2023. Data on microbiological identification and antimicrobial susceptibility were collected from multiple hospital areas, including the outpatient ambulatory ward (OUT), hospitalized in non-intensive care units (INX), intensive care units (ICU), and emergency rooms (EME). Clinical specimens with microbiological relevance were included, such as lower respiratory tract (LRT) samples (endotracheal aspirates and bronchoalveolar lavage), blood cultures, biopsies (joints, lymph node, bone, skin, lung, tissue, blood culture, muscle, liver), abscesses, urine, and sterile organic fluids (OF) that included ascitic, cerebrospinal, pleural, synovial, and pericardial fluids.

The bacterial species analyzed were *Escherichia coli*, *Klebsiella pneumoniae*, *Klebsiella aerogenes*, *Enterobacter cloacae*, *Pseudomonas aeruginosa*, *Acinetobacter baumannii*, *Stenotrophomonas maltophilia*, *Staphylococcus aureus*, *Enterococcus faecium*, and *Enterococcus faecalis*.

### 2.2 Microbiological identification and susceptibility testing

Bacterial identification was performed using the VITEK^®^ 2 (BioMérieux, Marcy l’ Etoile, France) and the MicroScan^®^ WalkAway^®^ (Siemens Healthcare Diagnostics, West Sacramento, CA). Databases generated by these systems were curated by a clinical microbiologist, retaining only isolates with an identification confidence greater than 95%. All datasets were encrypted to protect personal information and subsequently converted into WHONET format using the Backlink tool. Final analyses were conducted using WHONET 2025 (v25.11.6), thus, only one strain was included as recommended by Clinical and Laboratory Standards Institute (CLSI) M39 guidelines (Clinical, Laboratory Standards I, 2022). The antimicrobial panel tested varied according to the availability of routine panels at each site and the available instrument.

The antibiotics tested were: ceftazidime (CAZ), ceftriaxone (CRO), cefotaxime (CTX), cefepime (FEP), ceftaroline (CPT), aztreonam (ATM), ampicillin/sulbactam (SAM), ceftazidime/avibactam (CZA), ceftolozane/tazobactam (C/T), piperacillin/tazobactam (TZP), amikacin (AK), ciprofloxacin (CIP), levofloxacin (LVX), moxifloxacin (MFX), doripenem (DOR), ertapenem (ETP), imipenem (IPM), meropenem (MEM), trimethoprim/sulfamethoxazole (SXT), nitrofurantoin (NIT), fosfomycin (FOS), penicillin (PE), oxacillin (OXA), rifampicin (RIF), erythromycin (E), clindamycin (CC), daptomycin (DAP), linezolid (LZD), tetracycline (TE), ampicillin (AMP), high-load gentamycin (HLG). All abbreviations are presented in **Table 1**.

**Table 1:** Study abbreviations.

Abbreviations	Definition
AK	Amikacin
AMP	Ampicillin
AMP	Ampicillin
AMR	Antimicrobial Resistance
ATM	Aztreonam
C/T	Ceftolozane/Tazobactam
CAZ	Ceftazidime
CC	Clindamycin
CIP	Ciprofloxacin
CPT	Ceftaroline
CRO	Ceftriaxone
CTX	Cefotaxime
CZA	Ceftazidime/Avibactam
CZA	Ceftazidime/Avibactam
DAP	Daptomycin
DOR	Doripenem
E	Erythromycin
EME	Emergency Rooms
ESBL	Extended Spectrum ß-Lactamase
ETP	Ertapenem
FEP	Cefepime
FOS	Fosfomycin
HLG	High-Load Gentamycin
HLS	High-Load Streptomycin
I	Intermediate
INX	Non-ICU
IPM	Imipenem
LRT	Lower Respiratory Tract
LVX	Levofloxacin
LZD	Linezolid
MEM	Meropenem
MFX	Moxifloxacin
MRSA	Meticillin-Resistant Staphylococcus Aureus
NIT	Nitrofurantoin
OF	Organic Fluids
OUT	Outpatient Ambulatory Ward
OXA	Oxacillin
PE	Penicillin
R	Resistant
RIF	Rifampicin
S	Susceptible
SAM	Ampicillin/Sulbactam
SXT	Trimethoprim/Sulfamethoxazole
TE	Tetracycline
TZP	Piperacillin/Tazobactam

Cumulative antimicrobial susceptibility data were compiled following CLSI M39 (Clinical, Laboratory Standards I, 2022) recommendations, and clinical breakpoints were interpreted according to CLSI M100 (2025) (Clinical, Laboratory Standards I, 2025). Colistin results were excluded due to methodological limitations, and vancomycin testing in *S. aureus* was omitted.

### 2.3 Statistical analysis

A descriptive analysis was performed using frequency measures and percentages. Different susceptibility patterns between clinical specimens and clinical wards were compared using the χ^2^ test. *Post hoc* analyses were conducted using the exact Fisher test for antimicrobial agents with a *P* value <0.05. Comparisons by specimen type and wards were performed using a multiple-comparison adjustment based on the false discovery rate (FDR) method. All statistical analyses were performed using R Statistical Software Studio version 2025.5.0.496 (Boston, MA).

## 3 Results

### 3.1 Clinical specimens and overall antibiotic resistance

**Table 2** presents the distribution of samples by bacterial species and clinical specimen. Overall, the most common specimen types were urine (n= 4,247; 29.6%) and blood (n= 2,457; 17.1%).

**Table 2:** Sample distribution by bacteria and clinical specimen.

Microorganism	Overall	Biopsies and abscesses		Organic fluids		Lower respiratory tract		Blood		Urine		Other clinical specimen	
	n=	n=	%	n=	%	n=	%	n=	%	n=	%	n=	%
All bacteria	14,369	1,673	11.6%	1,208	8.4%	1,980	13.8%	2,457	17.1%	4,247	29.6%	2,805	19.5%
*E. coli*	5,737	625	10.9%	385	6.7%	194	3.4%	462	8.1%	3,100	54.0%	971	16.9%
*K. pneumoniae*	1,738	191	11.0%	151	8.7%	293	16.9%	485	27.9%	618	35.6%	-	0.0%
*E. cloacae*	624	82	13.1%	52	8.3%	116	18.6%	202	32.4%	72	11.5%	100	16.0%
*K. aerogenes*	76	-	0.0%	-	0.0%	-	0.0%	-	0.0%	-	0.0%	76	100.0%
*P. aeruginosa*	1,683	178	10.6%	169	10.0%	518	30.8%	294	17.5%	305	18.1%	219	13.0%
*A. baumannii*	1,437	104	7.2%	131	9.1%	426	29.6%	321	22.3%	101	7.0%	354	24.6%
*S. maltophilia*	333	-	0.0%	-	0.0%	170	51.1%	61	18.3%	-	0.0%	102	30.6%
*S. aureus*	1,407	266	18.9%	188	13.4%	263	18.7%	368	26.2%	51	3.6%	271	19.3%
*E. faecium*	342	60	17.5%	51	14.9%	-	0.0%	83	24.3%	-	0.0%	148	43.3%
*E. faecalis*	992	166	16.7%	81	8.2%	-	0.0%	181	18.2%	-	0.0%	564	56.9%

By microorganism, the most frequently isolated species was *E. coli* (n= 5,737), followed by *K. pneumoniae* (n= 1,738). Among Gram-negative bacteria, *P. aeruginosa* (n= 1,683) and *A. baumannii* (n= 1,437) were also highly represented, whereas *S. aureus* (n= 1,407) was the most frequently isolated Gram-positive organism.

*E. coli* was mainly associated with urine samples (54.0%), while *K. pneumoniae* also showed higher proportions in urine (35.6%) and blood (27.9%). *P. aeruginosa* was most frequently found in lower respiratory tract samples (30.8%), as was *A. baumannii* (29.6%). For *S. aureus* and *E. cloacae*, the most common specimen types were blood (26.2% and 32.4%, respectively).

**Table 3** shows the distribution of samples by bacterial species and hospital ward. Overall, the largest proportion of samples was obtained from non-ICU inpatient wards (41.3%). By bacterial species, *K. pneumoniae* showed the highest proportion of isolates from non-ICU inpatient wards (45.7%), as did *P. aeruginosa* and *A. baumannii*, which were also most frequently isolated in this setting (44.7% and 42.2% respectively).

**Table 3:** Sample distribution by bacteria and ward where sample was collected.

Microorganism	Overall	Outpatient		Emergency room		Inpatient (non-ICU)		ICU		Other Ward	
	n=	n=	%	n=	%	n=	%	n=	%	n=	%
All bacteria	14,369	342	2.4%	1,150	8.0%	5,932	41.3%	1,907	13.3%	5,038	35.1%
*E. coli*	5,737	204	3.6%	663	11.6%	1,971	34.4%	276	4.8%	2,623	45.7%
*K. pneumoniae*	1,738	49	2.8%	140	8.1%	795	45.7%	304	17.5%	450	25.9%
*E. cloacae*	624	-	0.0%	32	5.1%	312	50.0%	101	16.2%	179	28.7%
*K. aerogenes*	76	-	0.0%	-	0.0%	60	78.9%	-	0.0%	16	21.1%
*P. aeruginosa*	1,683	40	2.4%	137	8.1%	752	44.7%	492	29.2%	262	15.6%
*A. baumannii*	1,437	-	0.0%	33	2.3%	606	42.2%	230	16.0%	568	39.5%
*S. maltophilia*	333	-	0.0%	-	0.0%	185	55.6%	123	36.9%	25	7.5%
*S. aureus*	1,407	49	3.5%	71	5.0%	562	39.9%	185	13.1%	540	38.4%
*E. faecium*	342	-	0.0%	-	0.0%	220	64.3%	53	15.5%	69	20.2%
*E. faecalis*	992	-	0.0%	74	7.5%	469	47.3%	143	14.4%	306	30.8%

Overall, approximately half of the *E. coli* isolates were ESBL-producers, while carbapenem resistance remained low (1.4% for DOR and MEM, 1.7% for IPM, and 2.0% for ETP). In contrast, *K. pneumoniae* showed a 39.7% ESBL production and higher carbapenem resistance (16.7% for DOR and 14.8% for MEM).

Among non-fermenting bacteria, *A. baumannii* exhibited markedly higher resistance rates, with 87.8% resistant to IPM, compared with *P. aeruginosa*, which showed a resistance rate of 28.3% to the same antibiotic. Among Gram-positive organisms, 13.6% of *S. aureus* isolates were MRSA, all of which were susceptible to CPT. *E. faecium* showed high resistance to AMP (78.2%) and moderate resistance to VAN (23.9%) (see **Figure 1**; **Supplementary Figure S1**).

**Figure 1:**
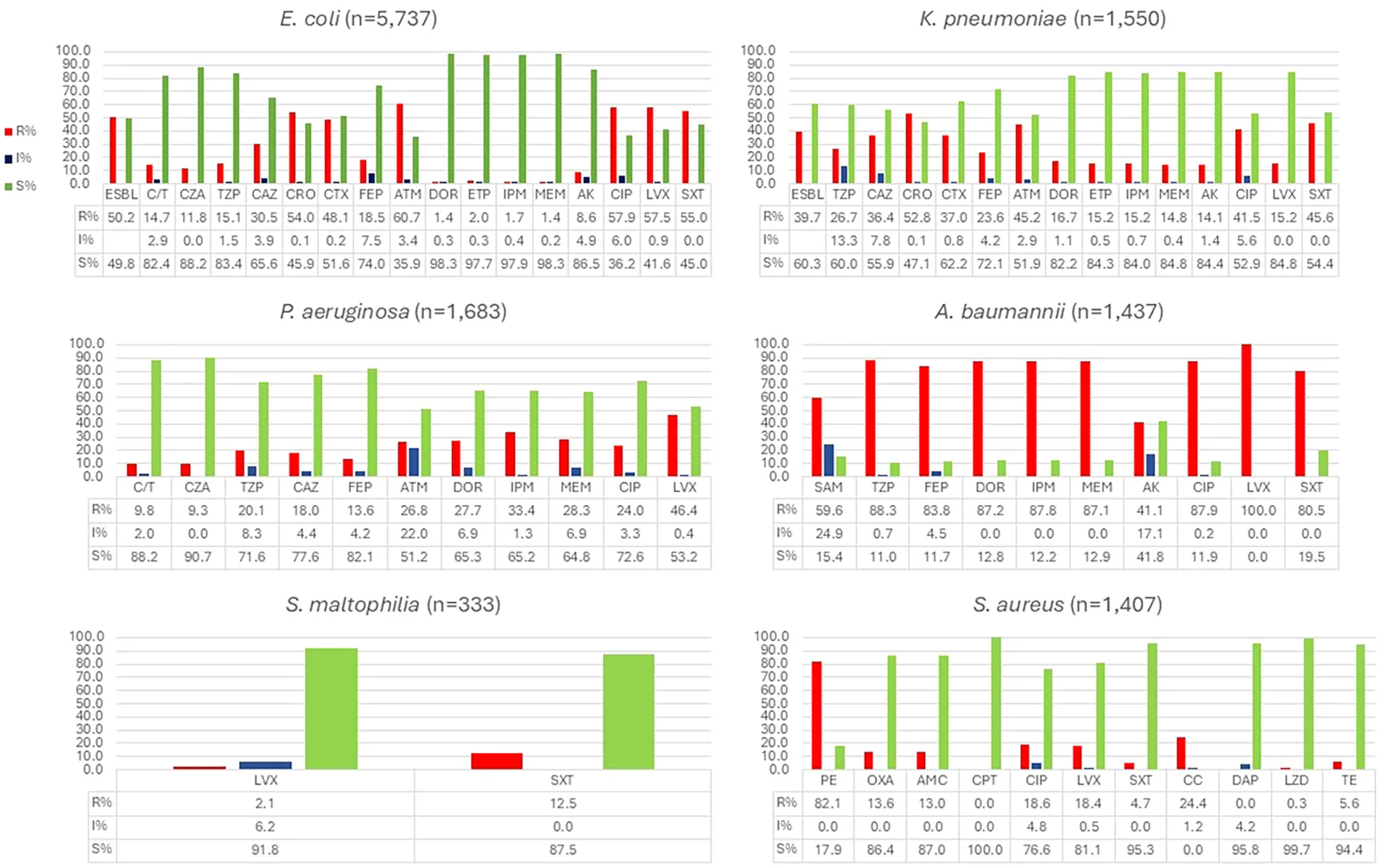
Susceptibility patterns from all microorganisms recovered.

### 3.2 Antibiotic resistance according to clinical specimens

#### 3.2.1 Biopsies and abscesses

Susceptibility patterns of microorganisms recovered from biopsies and abscesses are shown in **Figure 2**. *E. coli* showed a 58% rate of ESBL production, with high resistance to fluoroquinolone (67.4% to CIP and 59.4% to LVX), while carbapenem resistance remained low (5.6% to ERT).

**Figure 2:**
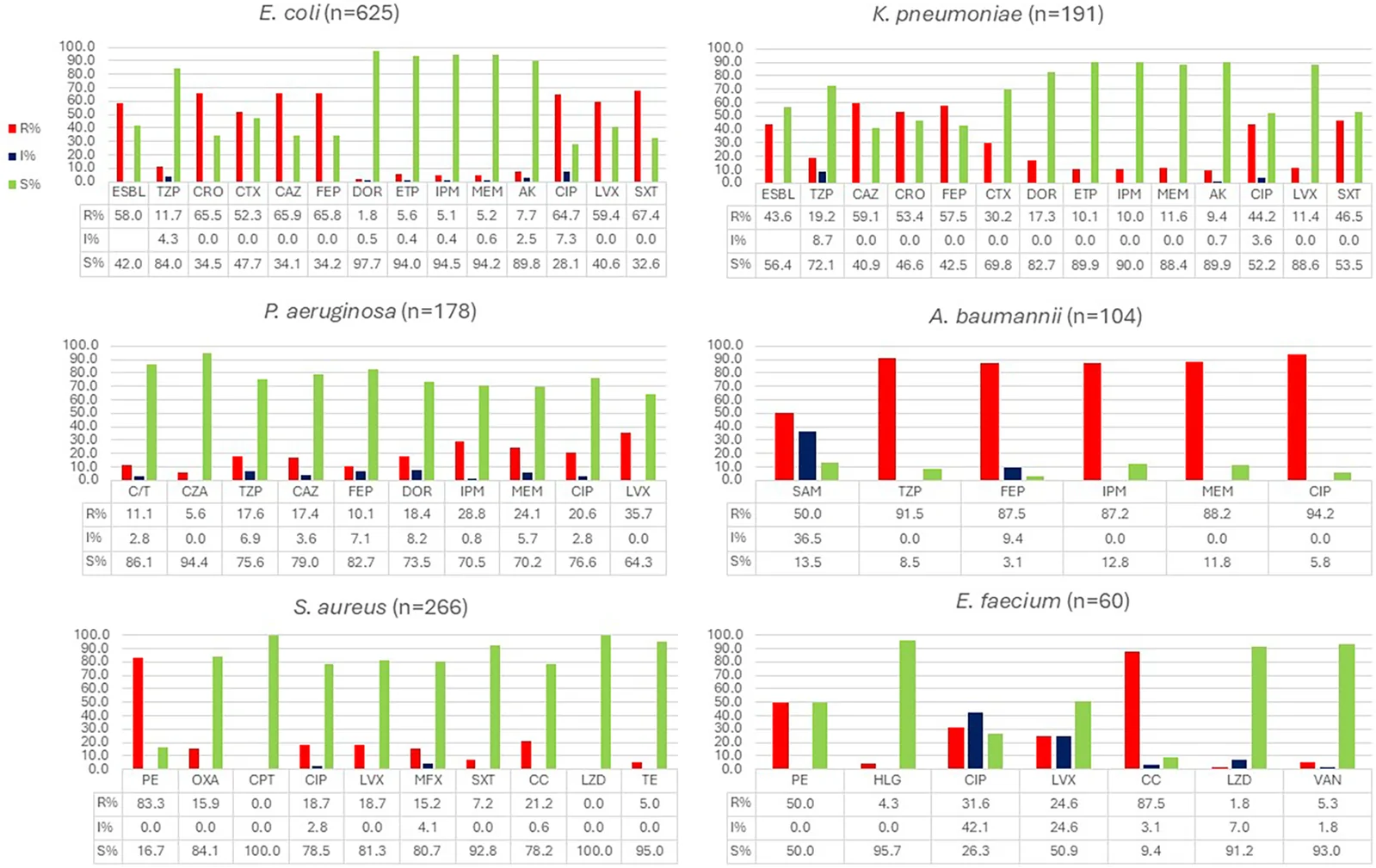
Susceptibility patterns from microorganisms recovered from biopsies and abscesses.

Among Enterobacterales, *E. cloacae* exhibited the highest carbapenem resistance (18.6% to ETP), whereas *K. pneumoniae* showed a 43.6% rate of ESBL production and 11.6% resistant to MEM.

Among non-fermenting bacteria, 88.2% of *A. baumannii* isolates were resistant to MEM. Among Gram-positive bacteria, MRSA accounted for 15.9% of *S. aureus*, while VAN resistance in *E. faecium* was low (5.3%) (see **Supplementary Figure S2**).

#### 3.2.2 Organic fluids

In OF, susceptibility patterns of recovered microorganisms are presented in **Figure 3**. ESBL production remained high in OF specimens for *E. coli* (52.2%) and *K. pneumoniae* (46.3%), with low carbapenem resistance observed in *E. coli* (1.8% to DOR).

**Figure 3:**
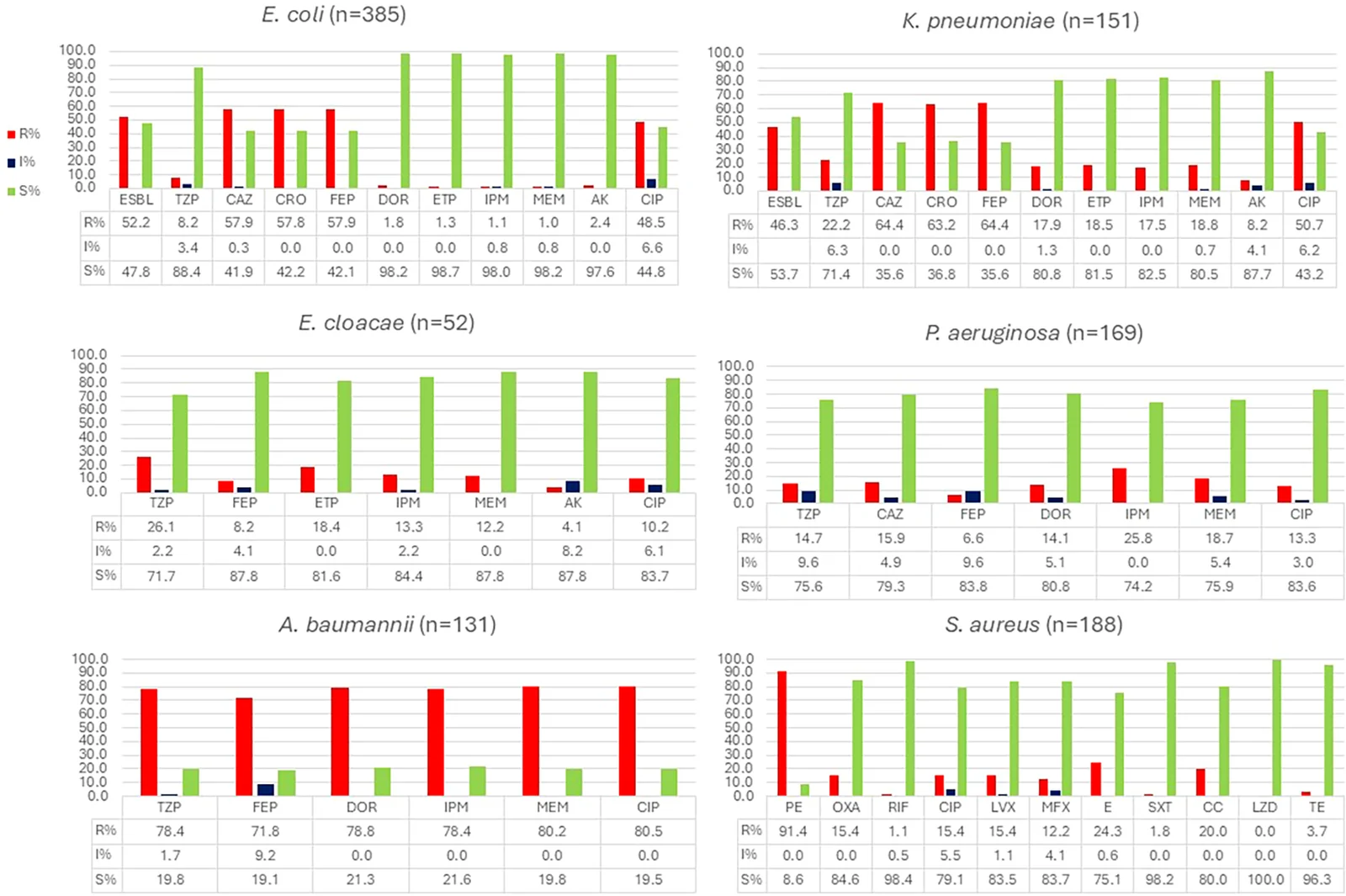
Susceptibility patterns from microorganisms recovered from organic fluids.

For *A. baumannii*, resistance rates across all antibiotics evaluated were approximately 80%. *P. aeruginosa* exhibited moderate carbapenem resistance (25.8% to IPM) but remained largely susceptible to cephalosporins (83.8% and 79.3% to FEP and CAZ, respectively).

Among Gram-positive organisms, MRSA was observed in 15.4% of S. aureus isolates. *E. faecium* showed high resistance to ampicillin (69.6%) and moderate resistance to vancomycin (26.1%), whereas *E. faecalis* remained largely susceptible to ampicillin (1.2% resistance) (see **Supplementary Figure S2**).

#### 3.2.3 Lower respiratory tract

Susceptibility patterns from microorganisms recovered from LRT are presented in **Figure 4**. Non-fermenting bacteria exhibited the highest levels of AMR. For *P. aeruginosa*, resistance was highest to carbapenems and fluoroquinolones (61.3% to IMP, 53.8% to MEM, and 47.8% to CIP), while lower resistance was observed for FEP (36.5%).

**Figure 4:**
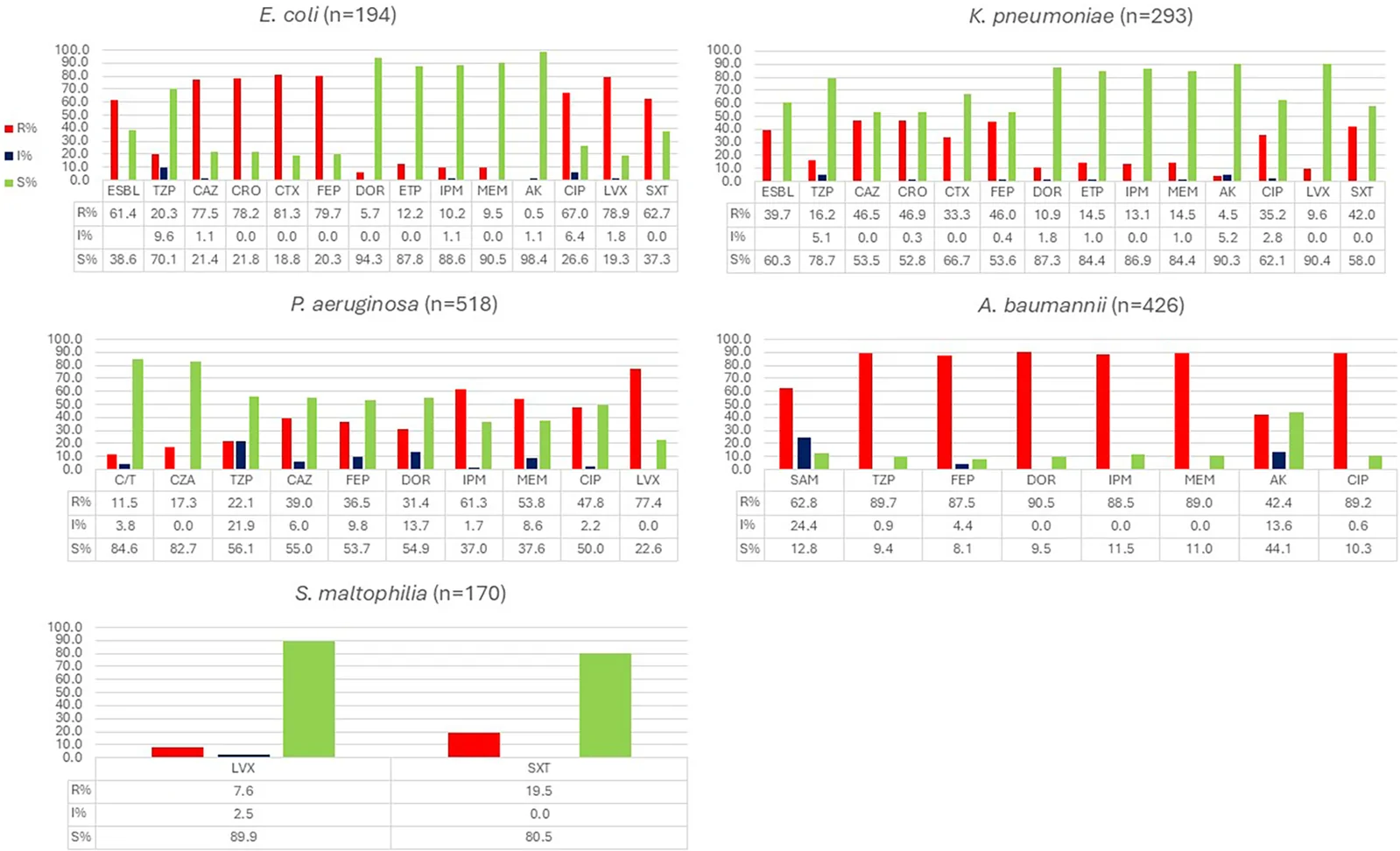
Susceptibility patterns from microorganisms recovered from lower respiratory tract.

For *A. baumannii*, carbapenem resistance was consistently high, ranging from 78.4% to 80.2% (78.4% to IMP, 78.8% to DOR, and 80.2% to MEM), while 42.4% of isolates were resistant to AK.

Among Enterobacterales, 46.3% of *K. pneumoniae* isolates were ESBL producers, while resistance to ERT remained comparatively lower (14.5%). *S. maltophilia*, showed low-to-moderate resistance, with 7.6% resistance to LVX and 19.5% to SXT.

Finally, among Gra-positive organisms, the proportion of MRSA was low (7.7%), and 99.2% of isolates remained susceptible to both CPT and LZD (see **Supplementary Figure S2**).

#### 3.2.4 Blood

In bloodstream isolates, susceptibility patterns are illustrated in **Figure 5**. ESBL production was frequent in both *K. pneumoniae* (35.4%) and *E. coli*, particularly in the latter (63.0%). However, carbapenem resistance remained low in E.coli (2.5% resistance to ERT) but was more pronounced in *K. pneumoniae* (23.2% resistant to ERT).

**Figure 5:**
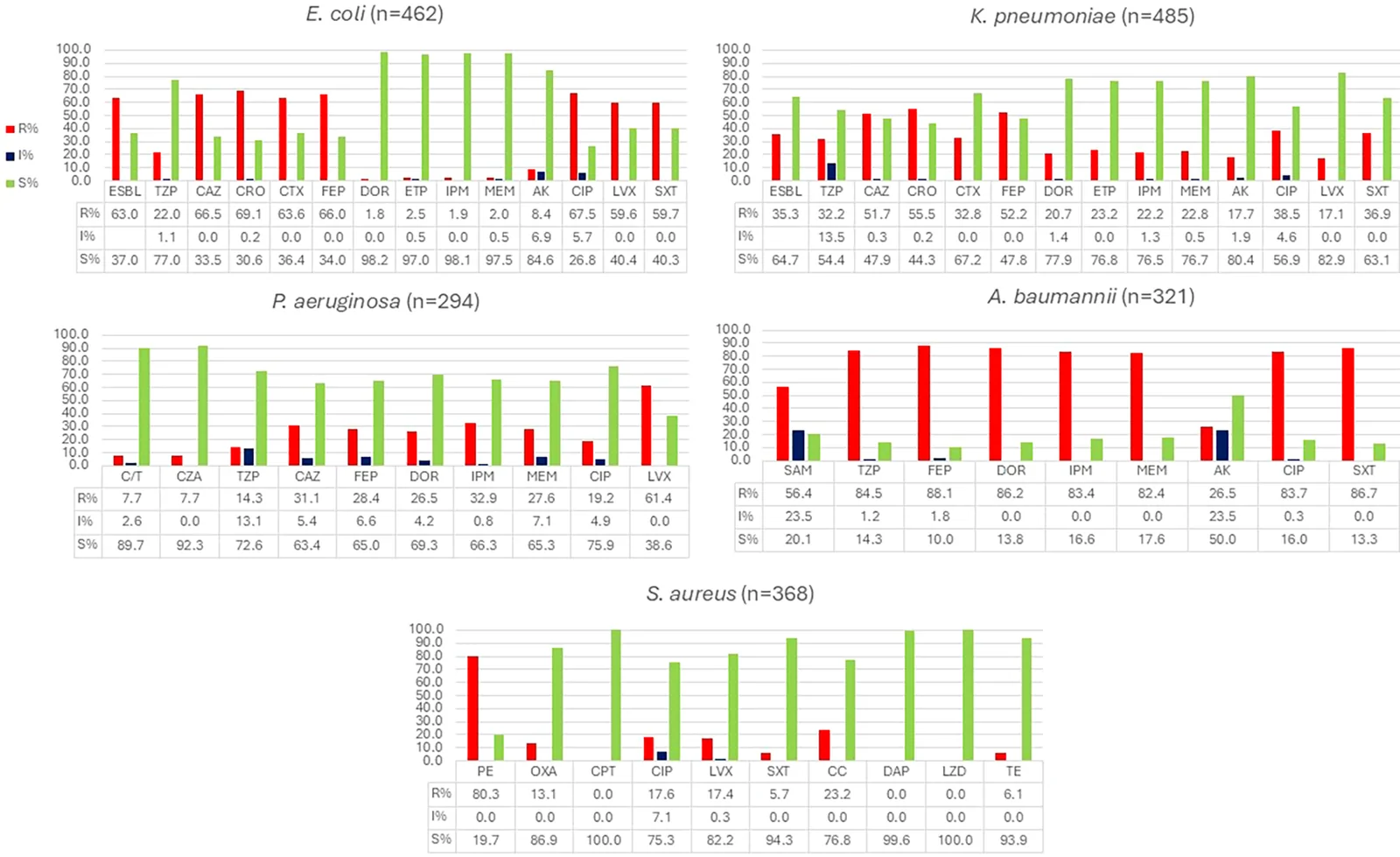
Susceptibility patterns from microorganisms recovered from bloodstream.

In contrast, *A. baumannii* exhibited high levels of carbapenem resistance, consistently exceeding 80% across agents (86.2% to DOR, 83.4% to IMP, and 82.4% to MEM) (see **Supplementary Figure S2**).

#### 3.2.5 Urine

Susceptibility patterns from microorganisms recovered from urine are shown in **Figure 6**. *E. coli* showed a high proportion of ESBL-producing strains (46.9%), while resistance to ERT remained low (1.2%). Most clinical isolates were susceptible to NIT and FOS (94.2% and 91.9% respectively).

**Figure 6:**
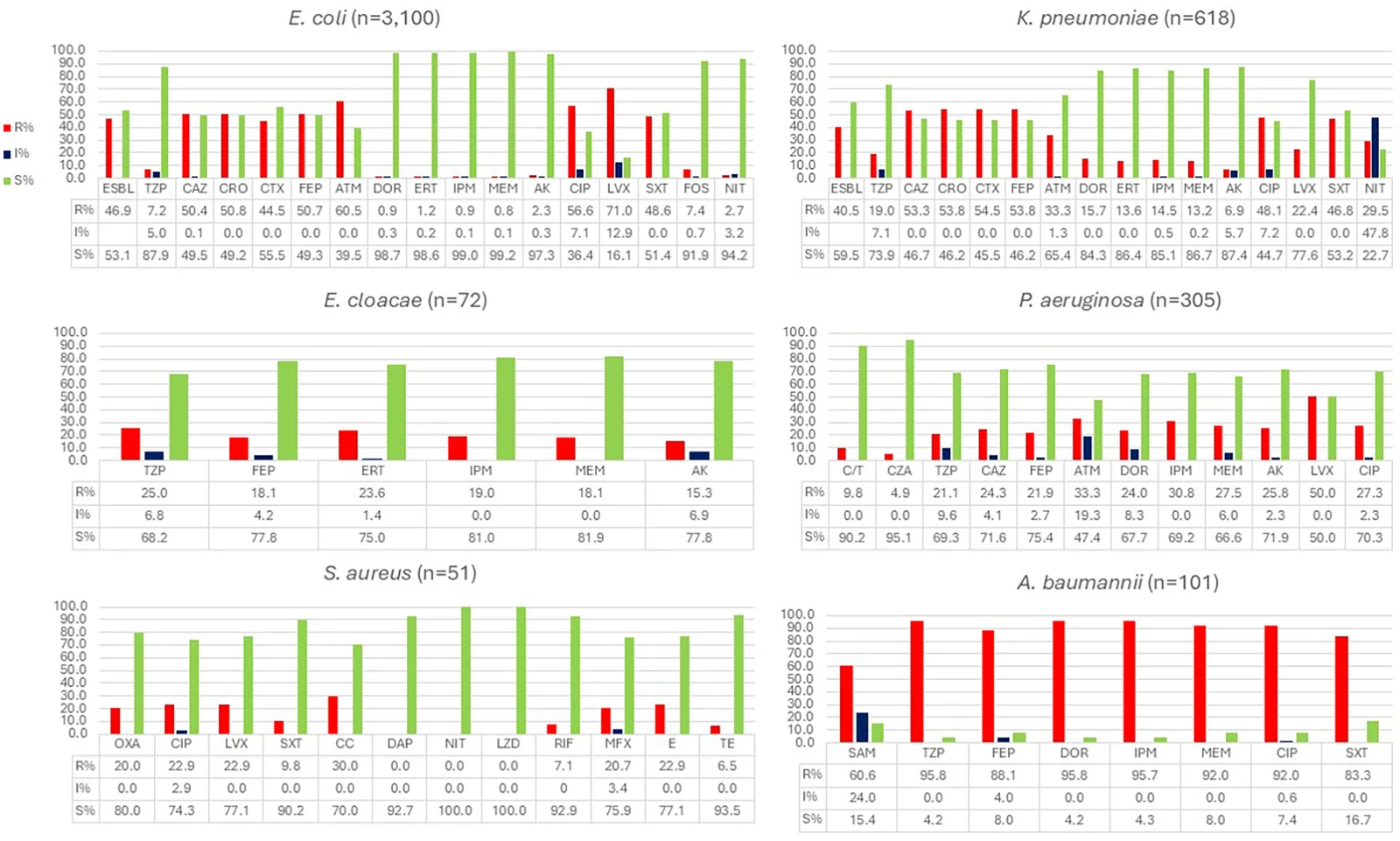
Susceptibility patterns from microorganisms recovered from urine.

*K. pneumoniae* exhibited a 40.5% prevalence of ESBL-producing strains, along with notable resistance to carbapenems (15.7% to DOR) and low-to-moderate susceptibility to NIT (22.7%) (see **Supplementary Figure S2**).

#### 3.2.6 Comparison of antibiotic resistance among clinical specimens

Resistance patterns varied according to specimen type. In *E. coli*, blood isolates showed the highest proportion of ESBL producers (p≤ 0.001), whereas isolates from the LRT exhibited the greatest resistance to carbapenems (DOR, p= 0.04; ETP, IPM, and MEM p≤ 0.001).

For *K. pneumoniae*, carbapenem resistance was higher in blood isolates (p< 0.005). Similarly, urine isolates of *E. cloacae* (p< 0.001)*, P. aeruginosa* (p< 0.001) and *A. baumannii* (p< 0.05) consistently showed higher carbapenem resistance compared with other specimen types.

No statistically significant differences were observed in *S. aureus* across specimen type. However, *E. faecium* isolates recovered from OF showed the highest resistance to VAN (p= 0.009). Raw data for each bacteria and antimicrobial tested by clinical specimen are available in **Supplementary Table S1**.

### 3.3 Resistance in hospital wards

#### 3.3.1 Outpatients

Among outpatients, susceptibility patterns are shown in **Figure 7**. In the outpatient setting, 43.6% of *E. coli* and *K. pneumoniae* isolates were ESBL producers. Carbapenem resistance remained low, observed in only 1.6% of *E. coli*, and 7.7% of *K. pneumoniae* isolates. More in depth data is available in **Supplementary Table S2**.

**Figure 7:**
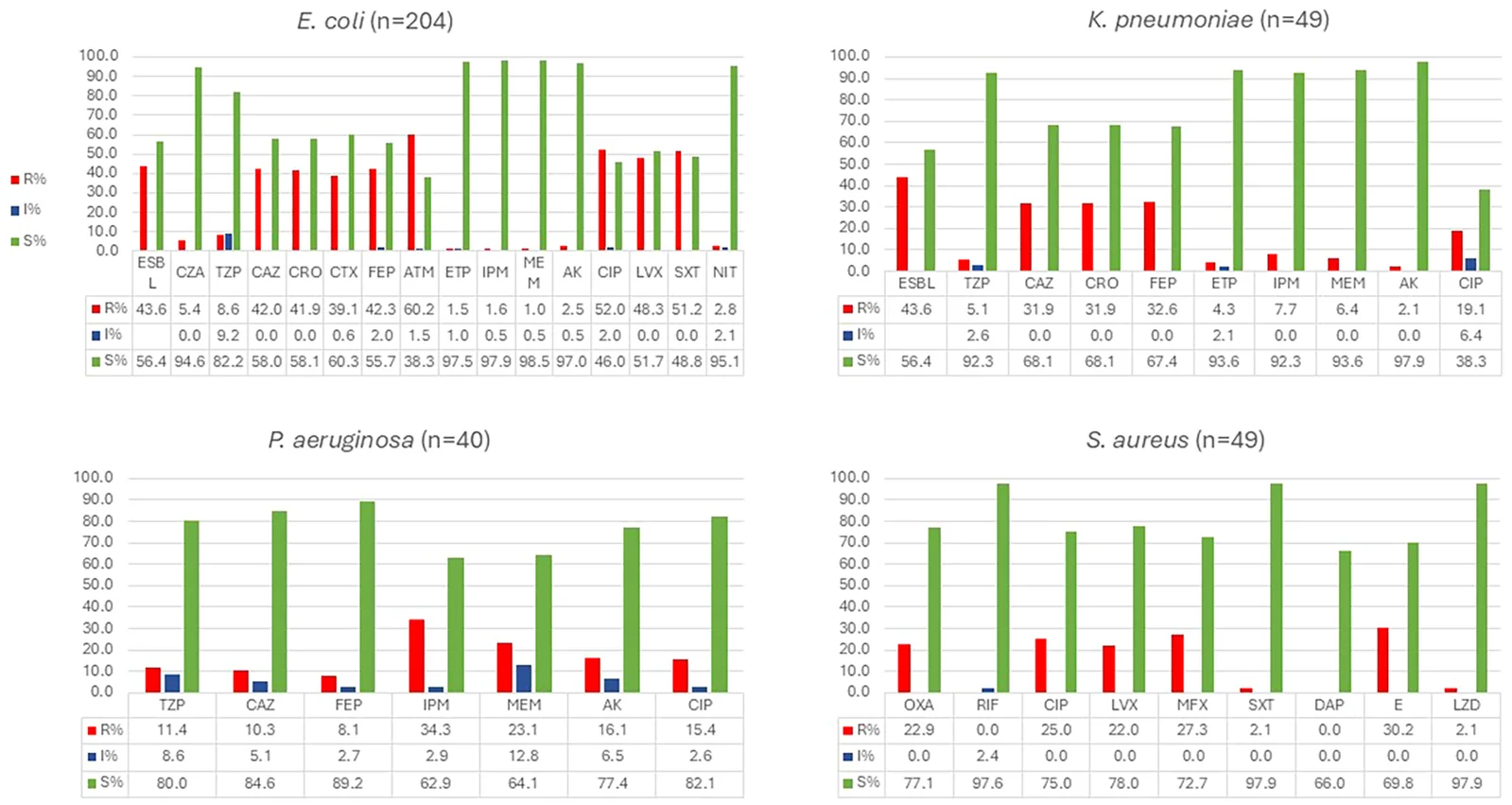
Susceptibility patterns from microorganisms recovered from outpatients.

#### 3.3.2 Emergency

Susceptibility patterns from microorganisms recovered from EME are shown in **Figure 8**. In the EME ward, 57.0% of *E. coli* were ESBL-producers, with notable resistance to CIP (58.9%), LVX (59.6%), and SXT (59.7%), while carbapenem resistance remained low (3.2%).

**Figure 8:**
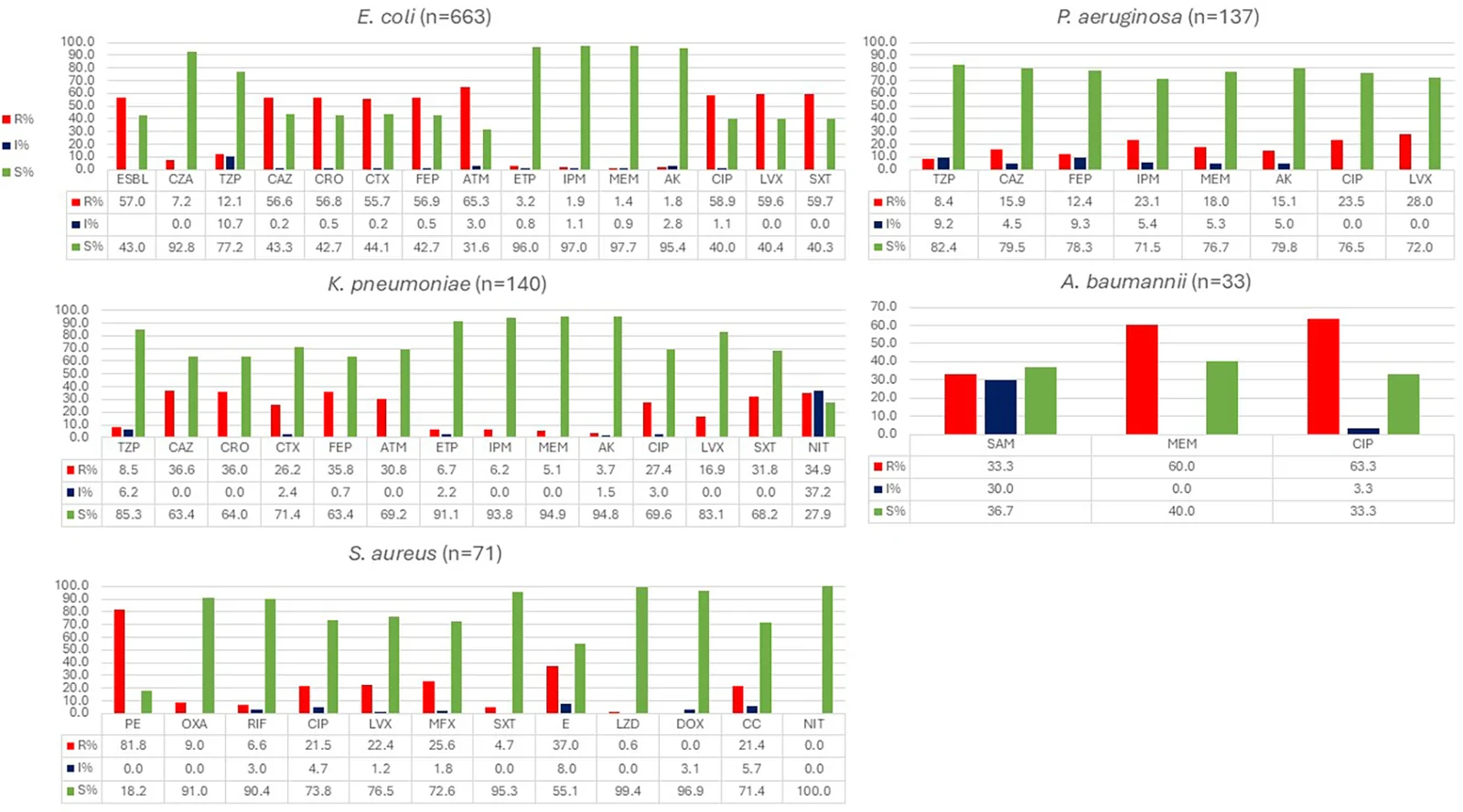
Susceptibility patterns from microorganisms recovered from emergency rooms.

Non-fermenting Gram-negative organisms showed moderate resistance levels, with *P. aeruginosa* displaying variability across β-lactams and carbapenems (15.9% to CAZ, 12.4% to FEP, 23.1% to IPM 18.0% to MEM), whereas *A. baumannii* showed elevated resistance to MEM (60%).

Among Gram-positive organisms, *S. aureus* exhibited moderate resistance to E (37.0%), MFX (25.6%), and LVX (22.4%). Data from *E. cloacae* and *E. faecalis* are available in **Supplementary Figure S3** and other bacteria in **Supplementary Table S2**.

#### 3.3.3 Hospitalized non-ICU (INX)

Susceptibility patterns from microorganisms recovered from non-ICU hospitalized patients are shown in **Figure 9**. In the INX ward, *E. coli* showed the highest proportion of ESBL-producing isolates (72.0%), while carbapenem resistance remained low (2.9% to ETP). *K. pneumoniae* exhibited a lower proportion of ESBL-producing isolates (23.3%) and moderate resistance to carbapenems (approximately 13.3%-13.8%).

**Figure 9:**
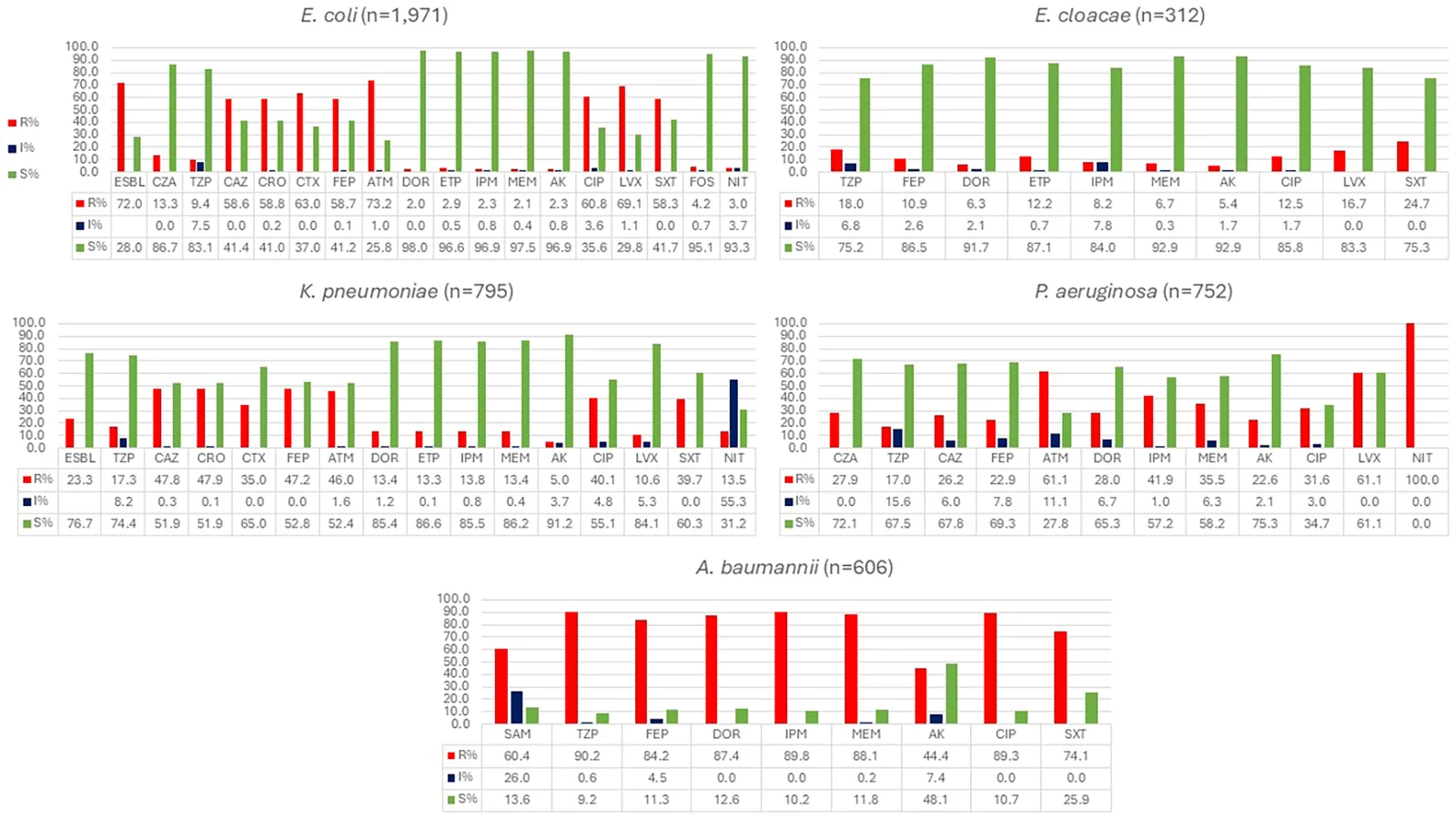
Susceptibility patterns from microorganisms recovered from non-Intensive care Unit hospitalized patients.

*E. cloacae* showed low-to-moderate resistance to carbapenems (6.3% to DOR, 6.7% to MEM, 8.2% to IPM and 12.2% to ETP).

Among non-fermenting organisms, *P. aeruginosa* exhibited moderate resistance to IPM (41.9%) and 26.2% to CAZ, while 72.1% of isolates remained susceptibility to CZA. In contrast, *A. baumannii* showed persistently high resistance to carbapenems, with 89.8% resistant to IPM. *S. maltophilia* showed 7.4% resistance to LVX and 14.9% to SXT (see **Supplementary Figure S3**).

Among Gram-positive organisms, *S. aureus* showed a moderate proportion of MRSA (12.8%) and high susceptibility to LZD (99.6%). *E. faecium* showed high resistance to AMP (67.5%), but low resistance to LZD (1.4%), while most *E. faecalis* isolates remained susceptible to LZD (87.6%) (see **Supplementary Figure S3**).

#### 3.3.4 Intensive care unit

In ICU isolates, susceptibility patterns are shown in **Figure 10**. Among Enterobacterales, *E. coli* and *K. pneumoniae* exhibited substantial resistance to cephalosporins and fluoroquinolones, along with moderate resistance to carbapenems. In *E. coli*, resistance was observed in 31.8% to CZA, 3.3% to DOR, 11.7% to ETP, and 66.8% to CIP. In *K. pneumoniae*, resistance reached 46.4% to CTX, 56.2% to FEP, 21.8% to ETP, and 19.3% to MEM (see **Supplementary Table S2**).

**Figure 10:**
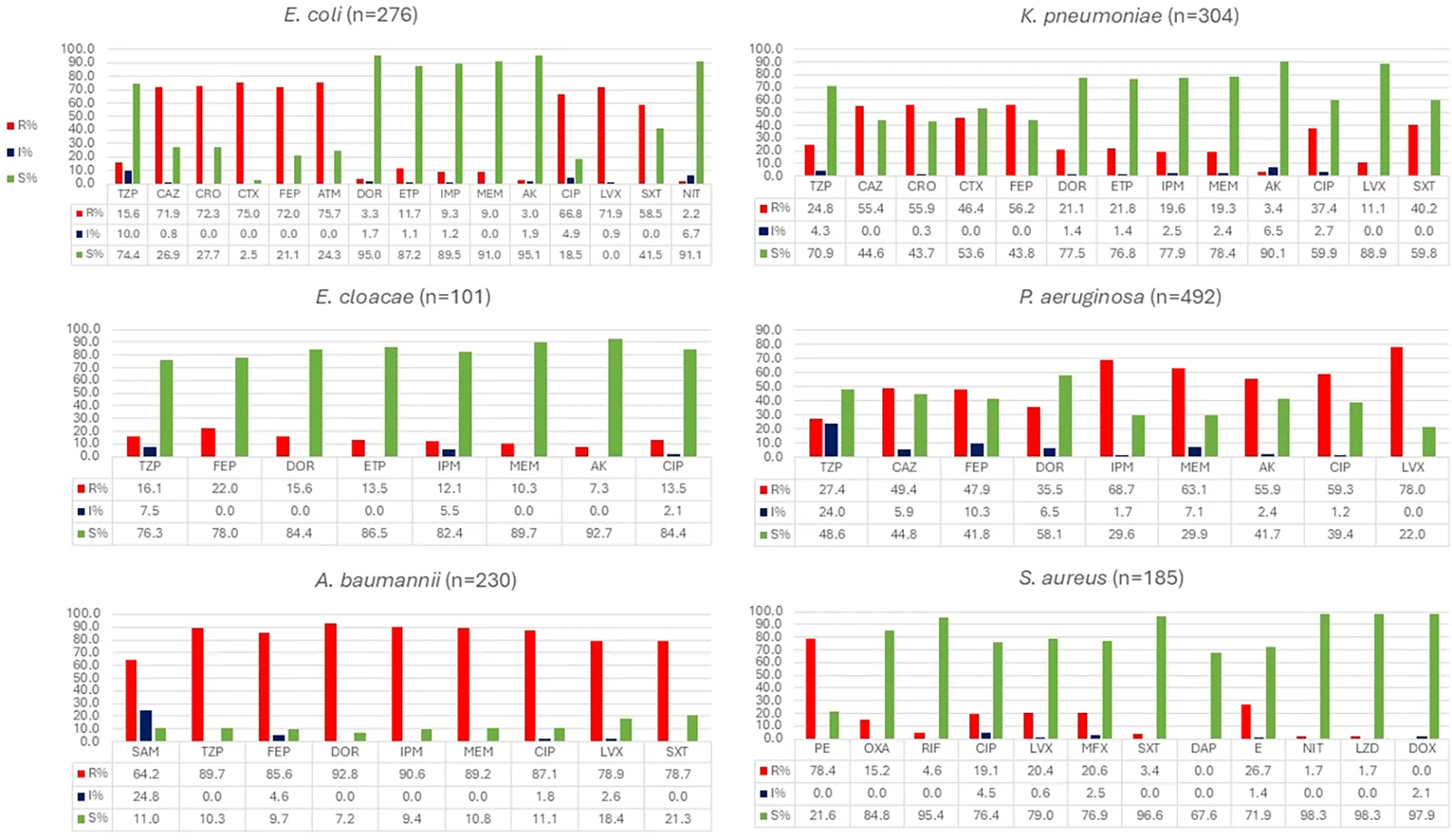
Susceptibility patterns from microorganisms recovered from ICU.

*E. cloacae* showed a similar pattern, with higher resistance to cephalosporins than to carpapenems (49.4% to CAZ and 22.0% to FEP vs 10.3% to MEM, 12.1% to IPM, 13.5% to ETP, and 15.6% to DOR) (see **Supplementary Table S3**).

Among non-fermenting organisms, *P. aeruginosa*, demonstrated high resistance to carbapenems (68.7% to IPM), while *A. baumannii* exhibited extremely high resistance across these agents (92.8% to DOR and 90.6% to IMP).

For *S. maltophilia*, resistance remained relatively low, particularly to fluoroquinolones (4.8% to LVX), although higher resistance was observed to SXT (19.8%).

For Gram-positive organisms, MRSA accounted for 15.2% of *S. aureus* isolates, with low resistance to LZD (1.7%). *E. faecium* showed notable resistance to LZD (13.5%) and VAN (11.5%) (see **Supplementary Figure S3**).

#### 3.3.5 Comparison of antibiotic resistance among clinical wards

Isolates from ICU patients showed the highest resistance rates across multiple species, particularly *K. pneumoniae, P. aeruginosa* and *A. baumannii* (all p< 0.001), as well as *E. cloacae* (FEP; p= 0.031), compared with isolates from other wards.

In contrast, isolates from the INX ward showed the highest resistance in *E. coli*, particularly to carbapenems (p<0.001), along with the highest frequency of ESBL-producing isolates (p= 0.007), compared with other wards.

Among Gram-positive organisms, *S. aureus* isolates from the EME department showed higher resistance overall (p= 0.009). *E. faecalis* isolates from the INX ward, demonstrated increased resistance to multiple agents, including NIT and LZD (both p< 0.001), PE (p= 0.008), and MFX (p< 0.001) (see **Supplementary Table S2**).

## 4 Discussion

### 4.1 ESBL-producing Enterobacterales

AMR has advanced rapidly and now represents one of the greatest threats to global health. The prevalence of resistant microorganisms continues to increase, while the development of new antimicrobial agents has slowed considerably. Resistance to carbapenems, in particular, has been associated with high mortality, further reinforcing the urgent need for coordinated global action. It is currently estimated that approximately 9% of all deaths are attributable to AMR (Minggui et al., 2022; Jinnethe et al., 2023).

The global prevalence of ESBL-producing Enterobacterales is estimated at 18.8% according to the ATLAS study, with an incidence of 15.2 cases per 1,000 population per year for ESBL-producing *E. coli* in North America (Ahmad et al., 2025). In our study, the overall proportion of ESBL-producing isolates was considerably higher (50.2%) than that reported in ATLAS. However, these findings are consistent with national data from Mexico (Colín-Castro et al., 2025), where the 2024 INVIFAR report documented ESBL production in 60.6% of *E. coli* and 48.6% of *K. pneumoniae* isolates. In contrast, we observed a lower proportion of ESBL-producing *K. pneumoniae* (39.7%).

The highest rates of ESBL production were observed among bloodstream isolates. Internationally, ESBL prevalence in bloodstream isolates has been reported as high as 85.4% in Ethiopia and 55.5% in China, while in Mexico, lower rates of 16.3% for *E. coli* and 26.9% for *K. pneumoniae* have been described (Jingjing et al., 2017; Dejenie Shiferaw et al., 2019; Viridiana et al., 2021). In our study, 63.0% of *E. coli* bloodstream isolates were ESBL producers, which are typically associated with resistance to third-generation cephalosporins (Paterson and Bonomo, 2005; Keshi et al., 2019).

These findings underscore the importance of regional and local epidemiological studies, as AMR patters are strongly influenced by factors such as infection control practices, antimicrobial availability, and prescribing behaviors.

### 4.2 Carbapenem-resistant Enterobacterales

In our study, carbapenem resistance in *K. pneumoniae* reached 14.8% for MEM representing approximately one-fifth of isolates overall. Globally, carbapenem resistance in *K. pneumoniae* varies widely, ranging from 14.3% in the United States to over 70.6% in countries such as Greece (Lin et al., 2024).

Consistent with global trends, the highest rates of carbapenem resistance were observed in bloodstream isolates reaching up to 62.8% in some settings (Andrew et al., 2019). However, we observed higher resistance among isolates recovered from biopsies and abscesses, in contrast to most studies, which typically report the highest resistance in bloodstream isolates. Data on resistance in these specimen types remain limited (World Health Organization Antimicrobial Resistance D, 2025).

### 4.3 Non-fermenting bacteria

Both *P. aeruginosa* and *A. baumannii* demonstrated high levels of resistance. The global prevalence of carbapenem resistance in *P. aeruginosa* is estimated at approximately 34.7%, increasing to nearly 66% in Latin America (Ramatla et al., 2025). In Mexico, resistance rates of around 30% have been reported, with regional variability, including rates of 32.5% in the Bajio region (Nieto-Saucedo et al., 2023; Ramatla et al., 2025).

In our study, *P. aeruginosa* resistance reached 61.3% and 53.8% to IMP and MEM, respectively, particularly in LRT specimens. This is consistent with its the role as a major pathogen in ventilator-associated pneumonia (Yixuan et al., 2024).

Similarly, *A. baumannii* exhibited concerning resistance levels, with urinary isolates showing resistance rates as high as 92% to MEM. While this organism is more commonly associated with bloodstream infections, where carbapenem resistance rates of up to 80.4% have been reported (Balkhair et al., 2023), it is also a key pathogen in catheter-associated urinary tract infections, where biofilm formation contributes to persistence (Pour et al., 2011; Mumtaz et al., 2024).

Adhesins such as Abp1D and Abp2D play an important role in urinary tract colonization and infection (Hazen et al., 2023; Tamadonfar et al., 2023). Although virulence factors were not assessed in this study, their contribution to the it is important to persistence and pathogenicity of *A. baumannii*, particularly in nosocomial settings, should be acknowledged.

### 4.4 Gram-positive bacteria

Gram-positive organisms, including *S. aureus* and *Enterococcus* spp., were also identified. Although they were not the primary focus of this analysis, their presence highlights the need for comprehensive surveillance of both Gram-negative and Gram-positive pathogens in clinical settings.

### 4.5 COVID-19: pandemic impact

The study period (2021–2023) coincided with the COVID-19 pandemic, a context that may have influenced AMR patterns. Widespread empirical use of broad-spectrum antibiotics was reported during this time, often exceeding the prevalence of confirmed bacterial co-infections (Malik and Mundra, 2022; Langford et al., 2023). This likely increased selective pressure for resistant organisms.

Additionally, healthcare system strain, including prolonged hospitalizations, increased ICU admissions, and disruption of antimicrobial stewardship and infection control programs, may have facilitated the emergence and spread of resistant pathogens (Langford et al., 2023; Emdin et al., 2025). ICU settings during the pandemic have been associated with increased rates of multidrug-resistant organisms compared with the pre-pandemic period (Do et al., 2025). These factors should be considered when interpreting the findings.

### 4.6 Study limitations

This study has several limitations. First, clinical data such as comorbidities, antimicrobial exposure, and patient outcomes were not available, limiting assessment of factors associated with resistance. Second, although phenotypic resistance was evaluated, carbapenemase genotyping, was not performed. Third, variations in specimen collection practices across institutions may have influenced isolates distribution.

An additional limitation relates to the categorization of certain specimen types. Biopsy and abscess samples were analyzed as a single group, although they represent distinct clinical entities with potentially different microbiological profiles. While both specimen types originate from normally sterile sites and are processed using similar microbiological principles, this pooled approach may still obscure specimen-specific differences, particularly regarding anaerobic taxa and infection depth, and thus limit the clinical interpretability of resistance patterns within this category. Similarly, LRT specimens included both endotracheal aspirates and bronchoalveolar lavage samples, which differ in their diagnostic specificity. Although both specimen types underwent standardized laboratory quality assessment prior to processing – reducing, but not eliminating, the possibility that colonizing flora influenced the results – their inherent differences in sampling method and clinical interpretation may have contributed to variability in the observed resistance rates and should be considered when interpreting these findings.

Fifth, regarding statistical analysis, the use of the FDR method to adjust for multiple comparisons may have reduced the likelihood of type I errors; however, it does not fully account for dependence among tests and may increase the risk of type II errors, particularly in subgroup analyses with limited sample sizes.

Sixth, differences between participating hospitals could not be assessed due to pooled analyses, potentially masking inter-institutional variability. Finally, the findings reflect a specific geographic region and may not be generalizable to other settings.

### 4.7 Clinical and public health implications

The high prevalence of ESBL-producing Enterobacterales and carbapenem-resistant organisms observed in this study highlights the urgent need to strengthen antimicrobial stewardship programs and infection prevention strategies. Continuous local surveillance is essential to guide empirical therapy and detect emerging resistance trends.

The elevated resistance observed in specific specimen types, particularly respiratory and urinary isolates, underscores the importance of targeted interventions in high-risk settings such as intensive care units. Future studies incorporating molecular characterization and clinical data will be critical to better understand resistance mechanisms and inform effective treatment and control strategies.

## 5 Conclusions

In this study, bloodstream isolates showed the highest resistance rates, particularly for *E. coli* and *K. pneumoniae*, exceeding some previously reported national estimates. Carbapenem resistance was also notable in isolates from biopsies and abscesses, an understudied specimen type for which limited comparative data are available.

Among non-fermenting pathogens, *P. aeruginosa* exhibited the highest resistance in lower respiratory tract specimens, while *A. baumannii* showed markedly elevated resistance in urinary isolates.

Taken together, these findings highlight important specimen-specific differences in AMR epidemiology and underscore the need for continuous surveillance, early identification of high-risk pathogens, and strengthened antimicrobial stewardship strategies across healthcare settings.

## Data Availability

The original contributions presented in the study are included in the article/**Supplementary Material**. Further inquiries can be directed to the corresponding author.

## Supplementary material

Supplementary Figure 1 Overall susceptibility patterns from microorganisms recovered.

Supplementary Figure 2 Susceptibility patterns from microorganisms recovered from clinical specimens.

Supplementary Figure 3 Susceptibility patterns from microorganisms recovered from clinical wards.

## References

[B17] Abdelaziz Abdelmoneim S. Mohamed Ghazy R. Anwar Sultan E. Hassaan M. A. Anwar Mahgoub M. (2024). Antimicrobial resistance burden pre and post-COVID-19 pandemic with mapping the multidrug resistance in Egypt: a comparative cross-sectional study. Sci. Rep. 14, 7176. doi: 10.1038/s41598-024-56254-4 38531847 PMC10966009

[B26] Ahmad I.-M. Bridgit M. Veronica A. Oladayo D. A. Ibukun M. A. Yemisi O. A. . (2025). Global distribution and incidence of multidrug resistant and ESBL producing Escherichia coli: an observational study of the ATLAS dataset. Clin. Epidemiol. Global Health 35, 102150. doi: 10.1016/j.cegh.2025.10215038826717

[B10] Aiesh B. M. Natsheh M. Amar M. Abutaha S. Qadi M. Abutaha A. . (2024). Epidemiology and clinical characteristics of patients with healthcare-acquired multidrug-resistant Gram-negative bacilli: a retrospective study from a tertiary care hospital. Sci. Rep. 14 (1), 3022. doi: 10.1038/s41598-024-53596-x 38321071 PMC10847101

[B34] Andrew J. S. Kalisvar M. Sharmila S. Arthur A. Maisra E.-B. Maria J. C. . (2019). Effect of carbapenem resistance on outcomes of bloodstream infection caused by Enterobacteriaceae in low-income and middle-income countries (PANORAMA): a multinational prospective cohort study. Lancet Infect. Dis. 19, 601–610. doi: 10.1016/s1473-3099(18)30792-8 31047852

[B39] Balkhair A. Saadi K. A. Adawi B. A. (2023). Epidemiology and mortality outcome of carbapenem- and colistin-resistant Klebsiella pneumoniae, Escherichia coli, Acinetobacter baumannii, and Pseudomonas aeruginosa bloodstream infections. IJID Regions 7, 1–5. doi: 10.1016/j.ijregi.2023.01.002 36936715 PMC10014253

[B11] Bezabih Y. M. Bezabih A. Dion M. Batard E. Teka S. Obole A. . (2022). Comparison of the global prevalence and trend of human intestinal carriage of ESBL-producing Escherichia coli between healthcare and community settings: a systematic review and meta-analysis. JAC-Antimicrob. Resist. 4 (3), dlac048. doi: 10.1093/jacamr/dlac048 35668909 PMC9160884

[B18] Çapar A. Özyiğitoğlu D. Başlılar Ş. (2025). Investigation of antibiotic resistance against pathogens isolated from respiratory samples in intensive care units after SARS-CoV-2 pandemic. BMC Infect. Dis. 25 (1), 1691. doi: 10.1186/s12879-025-12140-6 PMC12676884 41339825

[B1] Center for Disease C Prevention (2024). Antimicrobial resistance in the environment and the food supply: causes and how it spreads. Available online at: https://www.cdc.gov/ (Accessed March 15, 2025).

[B4] Chinemerem Nwobodo D. Ugwu M. C. Oliseloke Anie C. Al‐Ouqaili M. T. Chinedu Ikem J. Victor Chigozie U. . (2022). Antibiotic resistance: the challenges and some emerging strategies for tackling a global menace. J. Clin. Lab. Anal. 36 (9), e24655. doi: 10.1002/jcla.24655 35949048 PMC9459344

[B22] Clinical, Laboratory Standards I (2022). nalysis and Presentation of Cumulative Antimicrobial Susceptibility Test Data. 6th ed. Wayne (PA): Clinical and Laboratory Standards Institute. CLSI guideline M39.

[B23] Clinical, Laboratory Standards I (2025). M100 Performance Standards for Antimicrobial Susceptibility Testing, 35th Ed.

[B27] Colín-Castro C. A. López-Jácome L. E. Rodríguez-García M. J. Garibaldi-Rojas M. Rojas-Larios F. Vázquez-Larios M. D. R. . (2025). Antibiotic resistance and carbapenemase surveillance: INVIFAR report 2024 (Q1). PloS One 20, e0319441. doi: 10.1371/journal.pone.0319441 40238844 PMC12002462

[B29] Dejenie Shiferaw T. Abebe Aseffa N. Melese Hailu L. Tesfaye Legesse B. Hiwot Ketema W. Kassu Desta T. (2019). Extended-spectrum beta-lactamase production and multi-drug resistance among Enterobacteriaceae isolated in Addis Ababa, Ethiopia. Antimicrobial Resistance Infection Control 8, 39. doi: 10.1186/s13756-019-0488-4 30815254 PMC6377715

[B9] De Oliveira D. M. Forde B. M. Kidd T. J. Harris P. N. Schembri M. A. Beatson S. A. Walker M. J. M. . (2020). Antimicrobial resistance in ESKAPE pathogens. Clin. Microbiol. Rev. 33 (3), 10–1128. doi: 10.1128/cmr.00181-19 32404435 PMC7227449

[B13] De Souza J. D’Espindula H. R. S. Ribeiro I. D. F. Gonçalves G. A. Pillonetto M. Faoro H. (2025). Carbapenem resistance in acinetobacter baumannii: mechanisms, therapeutics, and innovations. Microorganisms 13, 1501. doi: 10.3390/microorganisms13071501 40732008 PMC12299412

[B47] Do D.-A. Duy-Nguyen V.-M. Nguyen T.-H. Le D-T-S. Nguyen H.-N. Dang D.-N. (2025). Antibiotic resistance among ICU patients during the COVID-19 pandemic and its associated factors: a retrospective study using electronic medical records in two Vietnamese hospitals. Epidemiol. Infection 153, 1–29. doi: 10.1017/s0950268825100307 40671598 PMC12394013

[B46] Emdin F. Orubu E. S. F. Rogers Van Katwyk S. Strong K. Shaver N. Abdullah K. . (2025). COVID-19 impact on AMR: a rapid scoping review, equity analysis and evidence gap map study. BMJ Global Health 10, e018118. doi: 10.1136/bmjgh-2024-018118 41320199 PMC12666195

[B12] Fernandez M. Gavaghan M. B. Al-Taie A. Stone G. Kotb R. E. M. (2023). Epidemiology of extended spectrum beta-lactamase (ESBL)-producing enterobacterales and antimicrobial resistance in urinary tract infection (UTI) and acute pyelonephritis (AP). Open Forum Infectious Diseases 10 (Supplement_2), ofad500-2437. US: Oxford University Press.

[B19] Ge M. Qiang W. Wang P. (2026). Emerging resistance to imipenem-relebactam among enterobacterales and non-fermenting gram-negative bacilli: a systematic review and meta-analysis. Infection 54, 1105–1116. doi: 10.1007/s15010-026-02739-5 41661521

[B42] Hazen J. E. Di Venanzio G. Hultgren S. J. Feldman M. F. (2023). Catheterization of mice triggers resurgent urinary tract infection seeded by a bladder reservoir of Acinetobacter baumannii. Sci. Transl. Med. 15 (678), eabn8134. 36630484 10.1126/scitranslmed.abn8134 PMC10464790

[B28] Jingjing Q. Dongdong Z. Lilin L. Yan C. Jiancang Z. Yan J. . (2017). High prevalence of ESBL-producing Escherichia coli and Klebsiella pneumoniae in community-onset bloodstream infections in China. J. Antimicrob. Chemother. 72, 273–280. 10.1093/jac/dkw372 27624571

[B25] Jinnethe R. Lauren K. Liang C. Lizhao G. Blake M. H. Eric C. . (2023). Global epidemiology and clinical outcomes of carbapenem-resistant Pseudomonas aeruginosa and associated carbapenemases (POP): a prospective cohort study. Lancet Microbe 4, e159–ee70. doi: 10.1016/s2666-5247(22)00329-9 36774938 PMC10016089

[B7] Kelly J. B. Nolan A. C. Zeden M. S. (2024). How can we escape the ESKAPEs: antimicrobial resistance mechanisms and what lies ahead? PloS Pathog. 20, e1012270. doi: 10.1371/journal.ppat.1012270 38870133 PMC11175505

[B31] Keshi L. Weiwei X. Shoulin L. Xiaodong L. Hao W. Junhai J. . (2019). Analysis of drug resistance of extended-spectrum beta-lactamases-producing Escherichia coli and Klebsiella pneumoniae in children with urinary tract infection. Saudi Med. J. 40, 1111–1115. doi: 10.15537/smj.2019.11.24547 31707407 PMC6901762

[B44] Langford B. J. So M. Simeonova M. Leung V. Lo J. Kan T. . (2023). Antimicrobial resistance in patients with COVID-19: a systematic review and meta-analysis. Lancet Microbe 4, e179–ee91. doi: 10.2139/ssrn.4099404 PMC9889096 36736332

[B20] Lee Y.-L. Wang W.-Y. Ko W.-C. Hsueh P.-R. (2024). Global epidemiology and antimicrobial resistance of Enterobacterales harbouring genes encoding OXA-48-like carbapenemases: insights from the results of the Antimicrobial Testing Leadership and Surveillance (ATLAS) programme 2018–2021. J. Antimicrob. Chemother. 79, 1581–1589. doi: 10.1093/jac/dkae140 38758189

[B33] Lin X. C. Li C. L. Zhang S. Y. Yang X. F. Jiang M. (2024). The global and regional prevalence of hospital-acquired carbapenem-resistant Klebsiella pneumoniae infection: a systematic review and meta-analysis. In Open forum infectious diseases (Vol. 11, No. 2, p. ofad649). US: Oxford University Press. 38312215 10.1093/ofid/ofad649 PMC10836986

[B45] Malik S. S. Mundra S. (2022). Increasing consumption of antibiotics during the COVID-19 pandemic: implications for patient health and emerging anti-microbial resistance. Antibiotics 12, 45. doi: 10.3390/antibiotics12010045 36671246 PMC9855050

[B2] McEwen S. A. Collignon P. J. (2018). Antimicrobial resistance: a one health perspective. Microbiol. Spectr. 6 (2), 10–1128. 29600770 10.1128/microbiolspec.arba-0009-2017 PMC11633550

[B24] Minggui W. Michelle E. Liang C. Blake M. H. Yunsong Y. Zhengyin L. . (2022). Clinical outcomes and bacterial characteristics of carbapenem-resistant Klebsiella pneumoniae complex among patients from different global regions (CRACKLE-2): a prospective, multicentre, cohort study. Lancet Infect. Dis. 22, 401–412. doi: 10.1016/s1473-3099(21)00399-6 34767753 PMC8882129

[B40] Mumtaz A. Saleem U. Arif M. Batool N. (2024). Investigation of antibiotic resistance and biofilm formation ability of Acinetobacter baumannii isolated from urinary catheters. Pakistan J. Med. Sci. 40, 2643–2647. doi: 10.12669/pjms.40.11.8754 39634881 PMC11613351

[B3] Murray C. J. Ikuta K. S. Sharara F. Swetschinski L. Aguilar G. R. Gray A. . (2022). Global burden of bacterial antimicrobial resistance in 2019: a systematic analysis. Lancet 399, 629–655. doi: 10.1016/s0140-6736(21)02724-0 35065702 PMC8841637

[B14] Najafabadi M. K. Soltani R. (2024). Carbapenem-resistant acinetobacter baumannii and ventilator-associated pneumonia; epidemiology, risk factors, and current therapeutic approaches. J. Res. Pharm. Pract. 13, 33–40. doi: 10.4103/jrpp.jrpp_50_24 39830948 PMC11737613

[B37] Nieto-Saucedo J. R. López-Jacome L. E. Franco-Cendejas R. Colín-Castro C. A. Hernández-Duran M. Rivera-Garay L. R. . (2023). Carbapenem-resistant gram-negative bacilli characterization in a tertiary care center from El Bajio, Mexico. Antibiotics 12, 1295. doi: 10.3390/antibiotics12081295 37627715 PMC10451683

[B32] Paterson D. L. Bonomo R. A. (2005). Extended-spectrum β-lactamases: a clinical update. Clin. Microbiol. Rev. 18, 657–686. doi: 10.1128/cmr.18.4.657-686.2005 16223952 PMC1265908

[B41] Pour N. K. Dusane D. H. Dhakephalkar P. K. Zamin F. R. Zinjarde S. S. Chopade B. A. (2011). Biofilm formation by Acinetobacter baumannii strains isolated from urinary tract infection and urinary catheters. FEMS Immunol. Med. Microbiol. 62, 328–338. doi: 10.1111/j.1574-695x.2011.00818.x 21569125

[B36] Ramatla T. Nkhebenyane J. Lekota K. E. Thekisoe O. Monyama M. Achilonu C. C. . (2025). Global prevalence and antibiotic resistance profiles of carbapenem-resistant Pseudomonas aeruginosa reported from 2014 to 2024: a systematic review and meta-analysis. Front. Microbiol. 16. doi: 10.3389/fmicb.2025.1599070 40761277 PMC12318982

[B43] Tamadonfar K. O. Di Venanzio G. Pinkner J. S. Dodson K. W. Kalas V. Zimmerman M. I. . (2023). Structure–function correlates of fibrinogen binding by Acinetobacter adhesins critical in catheter-associated urinary tract infections. Proc. Natl. Acad. Sci. 120 (4), e2212694120. doi: 10.1073/pnas.2212694120 PMC9942807 36652481

[B15] Thacharodi A. Vithlani A. Hassan S. Alqahtani A. Pugazhendhi A. (2024). Carbapenem-resistant Acinetobacter baumannii raises global alarm for new antibiotic regimens. iScience 27. doi: 10.1016/j.isci.2024.111367 39650735 PMC11625361

[B30] Viridiana G.-M. Ulises G.-R. Enrique B.-M. Moisés N.-N. Katya Rocío F.-M. Maria del Carmen C.-P. . (2021). ESBL-producing Escherichia coli and Klebsiella pneumoniae from health-care institutions in Mexico. J. Chemother. 33, 122–127. doi: 10.1080/1120009X.2020.1861815 33357158

[B16] William R. M. Cesar A. A. (2024). ESKAPE pathogens: antimicrobial resistance, epidemiology, clinical impact and therapeutics. Nat. Rev. Microbiol. 22, 598–616. doi: 10.1038/s41579-024-01054-w 38831030 PMC13147291

[B5] World Health O Antimicrobial resistance ( World Health Organization). Available online at: https://www.who.int/ (Accessed March 15, 2025).

[B21] World Health Organization (2015). Global action plan on antimicrobial resistance (Geneva, Switzerland: World Health Organization).

[B6] World Health Organization (2017). WHO publishes list of bacteria for which new antibiotics are urgently needed. Geneva: World Health Organization. Available online at: https://www.who.int/news/item/27-02-2017-who-publishes-list-of-bacteria-for-which-new-antibiotics-are-urgently-needed.

[B35] World Health Organization Antimicrobial Resistance D (2025). Global Antimicrobial Resistance and Use Surveillance System (GLASS) Report 2025 (Geneva: WHO).

[B8] Xuemei Z. Cecilia Stålsby L. Xueshan S. Xiaoqian H. Hengjin D. (2019). Economic burden of antibiotic resistance in ESKAPE organisms: a systematic review. Antimicrobial Resistance Infection Control 8, 137. doi: 10.1186/s13756-019-0590-7 31417673 PMC6692939

[B38] Yixuan L. Jason A. R. Mikaela M. W. Abdullah Tarik A. Patrick N. A. H. Fekade B. S. (2024). The global epidemiology of ventilator-associated pneumonia caused by multi-drug resistant Pseudomonas aeruginosa: A systematic review and meta-analysis. Int. J. Infect. Dis. 139, 78–85. doi: 10.1016/j.ijid.2023.11.023 38013153

